# Trends in pulmonary embolism mortality rates by age group in the United States, 1999–2019

**DOI:** 10.1016/j.ahjo.2022.100103

**Published:** 2022-02-12

**Authors:** Ayla Cash, Abdul Mannan Khan Minhas, Vanessa Pasadyn, Salik Nazir, Robert W. Ariss, Rajesh Gupta

**Affiliations:** a University of Toledo College of Medicine and Life Sciences, 3000 Arlington Avenue, Toledo, OH 43614, USA; b Division of Cardiovascular Medicine, University of Toledo College of Medicine and Life Sciences, 3000 Arlington Avenue, Toledo, OH 43614, USA; c Forrest General Hospital, Department of Medicine, Hattiesburg, MS 39401, USA

**Keywords:** Pulmonary embolism, Age, Mortality, Venous thromboembolism

## Abstract

**Introduction:**

Acute pulmonary embolism (PE) is a major cause of mortality in the United States. Recent reports indicate that PE-related mortality rates have increased among individuals younger than 65 years old. It remains unclear whether this increase in PE-related mortality is evenly distributed. A narrowly focused and clinically meaningful age group analysis is necessary.

**Methods:**

Death certificate data from the Centers for Disease Control and Prevention Wide-Ranging Online Data for Epidemiologic Research database were examined to determine all-cause PE mortality trends from 1999 to 2019 among adults 25–39, 40–54, 55–69, 70–84, and ≥85 years old. The crude death rates for individual years and annual percentage change (APC) were calculated to determine trends.

**Results:**

PE-related mortality rates increased among those 25–39, 40–54, and 55–69. Among individuals 25–39 years old, death rate increased from 1.8 to 2.0 (APC 0.7 [95% confidence interval (CI) 0.2 to 1.1]) between 1999 and 2014 and continued to increase from 2.0 to 2.4 (APC 4.1 [95% CI 1.8 to 6.5]) between 2014 and 2019. Among those 40–54 years old, the crude death rate increased from 5.7 to 7.5 (APC 2.0 [95% CI, 1.6 to 2.5]) between 2007 and 2019. Among those 55–69 years old the crude death rate increased from 15.6 to 18.5 (APC 2.2 [95% CI, 1.9 to 2.5]) between 2010 and 2019. Recent death rates decreased or plateaued among individuals older than 70.

**Conclusions:**

Individuals younger than 70 years had increase in PE-related mortality between 1999 and 2019 with marked increase among those 25–39 years old.

## Highlights

• Pulmonary Embolism (PE) - related mortality has increased in the United States among younger and middle-aged adults.
• It is unclear if previously reported increases in PE-related mortality were evenly distributed among age subgroups.
• We found that individuals 25-39 years old had the highest increases in PE-related mortality between 1999 and 2019.
• Conversely, recent death rates decreased or plateaued among individuals older than 70.
• This research provides a foundation for future investigations targeting PE surveillance and diagnosis among young adults.

## 1 Introduction

Although it is well recognized that the risk of acute pulmonary embolism (PE) increases with age, PE remains a major cause of mortality among younger adults in the United States (US) [1]. Smoking, obesity, trauma, surgery, prolonged air travel, oral contraceptive use, hormone replacement therapy, pregnancy, and post-partum status are the most common risk factors for provoked PE among younger individuals [2], [3], [4], [5], while hereditary thrombophilia is a common risk factor for unprovoked PE among younger individuals [6], [7], [8], [9].

PE-related mortality improved between 1979 and 1998 in the US [10]. A recent report indicated a reversal in these historic trends in the US across all sex-race, regional, and age subgroup analyses over the past decade [11]. This study also found that PE-related mortality increased by 23% among adults aged 25–64 between 2008 and 2018 [11]. A second report confirmed this trend [12]. While these results are compelling, it is unclear whether this increase in PE-related mortality is evenly distributed among individuals of different age groups. Specifically, some PE risk factors including pregnancy and oral contraceptive use may require more narrow age-stratification. Here, we report PE mortality trends from 1999 to 2019 among adults 25–39, 40–54, 55–69, 70–84, and ≥85 years old.

## 2 Methods

### 2.1 Statistics

The Centers for Disease Control and Prevention Wide-Ranging Online Data for Epidemiologic Research (CDC WONDER) database was used to extract death certificate data. The Multiple Cause-of-Death Public Use Record were analyzed to determine PE-related cause of death as a contributing cause on nationwide death certificate records. This database has been used in prior studies to determine nationwide trends in PE-related mortality [11]. PE-related mortality was identified using the *International Classification of Disease, 10th Revision* code I26.0 & I26.9 among patients ≥25 years of age.

PE-related deaths and population sizes were extracted from 1999 to 2019. Race/ethnicity was defined as non-Hispanic (NH) White, NH Black or African American, Hispanic of Latino, NH American Indian or Alaskan Native, and NH Asian or Pacific Islanders. Age groups were defined as 25–39, 40–54, 55–69, 70–84, and ≥85 years old. Age-adjusted mortality rates (AAMR) were determined by standardizing deaths to the 2000 United States Standard Population as previously done [11]. The crude death rates for individual years were calculated by dividing the number of PE-related deaths by the corresponding U.S. population of that year and expressed as PE-related deaths per 100,000 population per year. The Joinpoint Regression Program (Joinpoint V 4.9.0.0, National Cancer Institute) was used to determine significant temporal changes in trends of mortality within the study period [13]. This program identifies significant changes among annual mortality trends over time through joinpoint regression which fits models of linear segments where significant temporal variation occurred. Annual percentage change (APC) with 95% confidence internals (CI) for the crude death rates was calculated for the line segments linking a joinpoint using the Monte Carlo permutation test. APCs were considered increasing or decreasing if the slope describing the change in mortality through the identified time interval was significantly different than zero using 2-tailed t-testing. Statistical significance was set at *P* ≤ 0.05.

### 2.2 Study approval

The study was exempt from approval by the Institutional Review Board at the University of Toledo due to utilization of deidentified, publicly available data.

## 3 Results

**Fig. 1:**
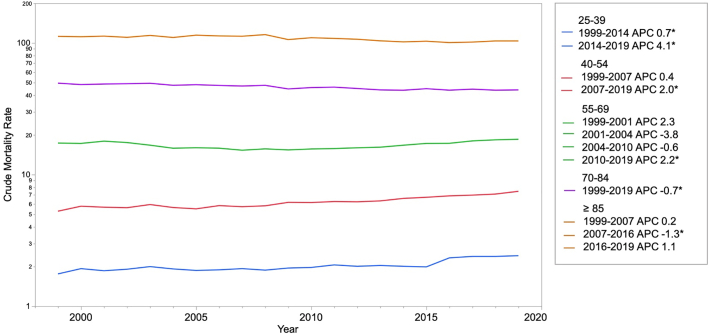
Age-stratified pulmonary embolism mortality rates in the United States, 2009 to 2019. *Indicates that the annual percentage change (APC) is significant different from zero at α = 0.05.

Among individuals 25–39 years old, the crude death rate increased from 1.8 to 2.0 (APC 0.7 [95% CI, 0.2 to 1.1]) between 1999 and 2014 and continued to increase from 2.0 to 2.4 (APC 4.1 [95% CI, 1.8 to 6.5]) between 2014 and 2019 (Fig. 1). Among those 40–54 years old, the crude death rate increased from 5.7 to 7.5 (APC 2.0 [95% CI, 1.6 to 2.5]) between 2007 and 2019. Among those 55–69 years old the crude death rate increased from 15.6 to 18.5 (APC 2.2 [95% CI, 1.9 to 2.5]) between 2010 and 2019. Among those 70–84 years old the crude death rate decreased from 49.5 to 44.0 (APC −0.7 [95% CI, −0.8 to −0.5]) between 1999 and 2019. While the crude death rate among those older than 85 decreased from 112.3 to 100.5 (APC −1.3 [95% CI, −1.9 to −0.7]) between 2007 and 2016, the crude death rate increased non-significantly among this cohort between 2016 and 2019.

Overall, males had higher AAMR than females (14.76 vs 13.24) throughout the study period. Among race/ethnic groups, NH Black people had highest AAMR (24.04) followed by NH White (13.75), NH American Indians or Alaskan Native (10.77), Hispanic or Latino (7.96), and NH Asian or Pacific Islander (4.16) people.

## 4 Discussion

Our findings provide nationwide estimates of trends in PE-related mortality rates from 1999 to 2019 among patients aged 25–39, 40–54, 55–69, 70–84, and ≥85. Crude death rates increased significantly for individuals 25–39, 40–54, and 55–69 years old since 1999, 2007, and 2010 respectively. Among individuals older than 70, the death rate either decreased or plateaued during the study period. In addition, PE-related mortality was highest among NH Black people and males compared with other races/ethnicities and females, respectively, as previously described [11], [12].

Our findings are consistent with two recent studies that reported a recent increase in PE-related mortality among adults younger than 65 years old in the US [11], [12]. Notably, our study reports that individuals aged 25–39 years old have marked increase in APC for PE-related mortality (4.1%) during the study period, occurring between 2014 and 2019. The rising PE-related mortality rate among individuals aged 25–39 is particularly concerning.

It is unclear why PE-related mortality is increasing among young individuals in the US. In contrast, PE-related mortality in Canada has decreased among all age groups between 2000 and 2017 [12]. Since the study population is de-identified and cross-sectional, we were unable to examine the influence of risk factors including obesity and cigarette smoking, both of which are associated with increased risk of thrombosis [14], [15].

The strengths of this study include the utilization of a national database that captures all death certificates in the US and the novel reporting of PE-related mortality according to fine sub-stratification of age groups. In addition, our analysis included death certificates with any mention of PE, rather than PE listed as the underlying cause of death only. This allowed for a broader estimation of the PE burden. One limitation is that death certificates are subject to human error and could be inaccurate if miscoded.

## 5 Conclusion

PE-related mortality rates are increasing among individuals younger than 70 years of age in the US, with the highest relative increase in individuals aged 25–39 years old.

## Funding

This work was supported by funding from the 10.13039/100012569University of Toledo to the principal investigator (R.G.).

## Declaration of competing interest

The authors declare the following financial interests/personal relationships which may be considered as potential competing interests: Dr. Rajesh Gupta is a co-owner of Thermomorph LLC, an early-stage company developing a device for venous thromboembolism (DVT and PE) treatment.

All of the remaining authors have nothing to disclose pertaining to the current manuscript.
